# A Game of Covid: Strategic Thoughts About a Ludified Pandemic

**DOI:** 10.3389/fpsyg.2021.607309

**Published:** 2021-06-29

**Authors:** Marius Hans Raab, Niklas Alexander Döbler, Claus-Christian Carbon

**Affiliations:** ^1^Department of General Psychology and Methodology, University of Bamberg, Bamberg, Germany; ^2^Bamberg Graduate School of Affective and Cognitive Sciences (BaGrACS), Bamberg, Germany

**Keywords:** strategic gamification, COVID-19, pandemic, ludification, analogical reasoning, crisis

## Abstract

Many aspects of handling the COVID-19 pandemic bear a resemblance to patterns found in games. We observe point displays and leader boards, the visible assumption of roles, classic archetypes, the collection of resources, and spatial awareness. We argue that these patterns manifest spontaneously as a form of analogical reasoning, because people lack cultural and individual norms as well as cognitive scripts for a pandemic. Trying to find systematic similarities between a novel and a familiar situation is an essential cognitive strategy and a cultural tool, resulting in a spontaneous ludification of this crisis. Unfortunately, most institutions, the media and policymakers focus on attributes that are easy to communicate, not on relations and causal chains. This results in shallow analogies, where the mechanisms and dynamics of COVID-19 are not addressed. This can cause a sense of helplessness, where many people remain passive viewers. A pandemic, however, calls for cooperative action of people who understand the relations between different factors and stakeholders in order to mitigate several negative effects linked to such a crisis. We propose a psychologically founded “Strategic gamification” (here in the context of a pandemic), a form of sense-making that builds on spontaneously emerging ludic elements. By extending upon those elements through the lens of game design, we can shape the mechanics, dynamics and esthetics of a serious context in a more meaningful way. The resulting analogies have better predictive power and are suited to utilize positive aspects of gamification like engagement, elaboration and collaboration.

## Introduction

With a death toll of three million (effective April 16, 2021), and over 130 million infected people (WHO, 2021), the SARS-CoV-2 pandemic has had a devastating effect on families, communities and nations all around the globe. It has thoroughly shattered personal and professional routines everywhere. With the severity as well as the novelty of this situation, we argue that people are missing heuristics and strategies to cope and thus fall back to patterns of perception and action that are usually associated with games. As infants, we discover the world by playing. Novelty, complexity, ambivalence and danger are approached spontaneously (given that parents provide food and protection of serious harm), with alternating states of effort and relaxation. As the world becomes more familiar when growing up, rules provide a framework that novelty, complexity ambivalence and danger can be experienced again in a game; a reality as-it-were that is not fully known yet and allows for exploration (Heckhausen, 1964). Game and play are part of virtually everybody’s experience of growing up, of understanding the world and its dangers.

COVID-19 is indeed creating new challenges, such a pandemic is complex and dangerous, and it also shows ambivalence. We will argue that this results in a spontaneously *ludified* (Raessens, 2006) pandemic. We understand ludification here as a phenomenon where people pick up narrations (i.e., stories that explain causal and temporal relations and bear subjective meaning), game metaphors and game elements to construct identity and to better understand culture and society. This builds on the idea of Johan Huizinga’s *Homo Ludens* (Huizinga, 1949). Connecting this observation of the spontaneous application of game elements to COVID-19, this paper offers a perspective on how to capitalize on this phenomenon by a *strategic gamification*. This includes an inventory where “ludic elements or qualities, or non-game objects and experiences that use design elements from games” (Walz and Deterding, 2014, p. 7) are spontaneously applied by people in a non-game context. Building on these elements, a flexible plan with clear long-term goals can be devised, where game mechanisms and ludic ingredients foster sense-making by providing inspiration for analogical reasoning. Our essential goal here is: strengthening people’s motivation for a cooperative effort to effectively mitigate this pandemic.

## Drawing Analogies

COVID-19’s devastating potential is sometimes compared to the 1918 flu pandemic (that is better, but problematically known as Spanish Flu) that has killed tens of millions of people about a 100 years ago (Barro et al., 2020). The grave consequences of the 1918 flu pandemic are a haunting, but now grossly vague collective memory of a ravaging disease. The memory is vague, mostly accessible via history books, depicted in black-and-white photography or through collected stories. We lack valid and detailed knowledge of everyday-life impact, especially regarding psychological aspects, as well as details about countermeasures. Consequently, we fall short in drawing specific inferences on how to handle such a threat in our time.

Major economic crises are relatively frequent (with a recent global recession in 2008 and another one ramping up just now due to the pandemic). While the last global war ended in 1945, armed conflicts are still a recurrent phenomenon (like, e.g., in Iraq and Afghanistan with U.S. involvement; and with the Yugoslav Wars and the recent war in Ukraine/Donbas within Europe) and are thus present in our news cycle.

We clearly do not have comparable experience concerning the COVID-19 pandemic. Severe diseases so far have been rather delimited geographically (like SARS-1 2002 or Ebola), are not present in Western countries (e.g., Malaria), are not contagious in everyday contact (e.g., HIV), are seasonal, and have vaccines available (H1N1 and other influenza diseases), or are specific to certain treatment of food (EHEC). COVID-19 is global, present in Western countries, and very contagious. Even though vaccinations are now available, the manufacturing of vaccines and their distribution will take a long time to achieve herd immunity. Also, several new variants like B.1.1.7 (501Y.V1, presumably emerging from the United Kingdom) and B.1.351 (501Y.V2, presumably emerging from South Africa) raised concerns that vaccines approved in many countries since December 2020 might become less effective over time. Thus, COVID will keep being a prevalent topic.

There is no established cultural norm in North America and Europe to handle such a pandemic. Even on a level of science-driven disease control, clear roadmaps for a pandemic are missing. The *German Influenza Pandemic Preparedness Plan* (Robert Koch Institut, 2018, p. 215) states: “In summary, a gap of knowledge exists regarding the effectiveness of the non-pharmacological interventions presented here, so there is an urgent need for more research including high-quality studies.” We are observing the consequences of this knowledge gap right now; even more so as well-understood and scientifically evaluated behavioral measures going beyond hand hygiene could help: “In case of a severe pandemic, a combination of different non-pharmacological interventions can be an effective instrument to attenuate the pandemic impact.” (Robert Koch Institut, 2018, p. 215).

For individuals, there is no precedent for action: In terms of cognitive psychology, people lack the *scripts* (Tomkins, 1979) to accommodate for the shattered daily routines. School, shopping, personal hygiene, travel, and work: The *scenes*, speaking again with Tomkins (1979), are so different that appropriate responses are yet to be identified and developed. Script theory has already been applied to improve patient expectations and communications in medical settings (St.Amant, 2020): Specific props (e.g., an insurance card), roles (clerk, nurse, and doctor), and appropriate responses to medical objects (like a stethoscope) can guide our behavior during an emergency room visit. Usually, such scripts are learned and refined throughout a lifetime, by personal experience, and by observing behavior in movies and T.V (for aggressive scripts, see Huesmann, 1988).

When facing novel and dangerous challenges, analogical reasoning is a core cognitive mechanism (and cultural tool) to exploit previously acquired knowledge from another domain (Earley, 2003). Analogies work by identifying the similarity of attributes and relations between a novel/abstract/unfamiliar and an already known/concrete/familiar situation, allowing for inferences, and reducing uncertainty (Chan et al., 2012).

Analogies can be built upon the relational structure of a situation (Gentner, 1989). A very well-known example is the electronic–hydraulic analogy, where the relations between voltage, current and charge are explained by a fluid system with pipes, pumps and valves. Everyday experience with simple hydraulic systems (e.g., the plumbing in a house) can be transferred to the domain of electricity. As the relations between pressure, flow and quantity are mapped in a meaningful way onto voltage, current and charge, this analogy allows for a rather good understanding of an invisible and hard-to-understand phenomenon.

Another way to build an analogy is to focus on rather superficial similar attributes. Novices in a domain resort to mere appearance or literal similarity with a higher probability when transferring knowledge to a new situation. This might result in misguided inferences or incorrect predictions that lack understanding of the key causal principles (Gentner, 1989). “The mitochondria is the powerhouse of the cell,” for example, is a superficial analogy. A powerhouse uses open combustion or nuclear fission to generate steam, which drives a generator, which produces electricity. A mitochondrion is so different in its chemical processes and its inner relations that imagining a “powerhouse” does not add any deeper understanding.

## A Ludified Pandemic

**FIGURE 1:**
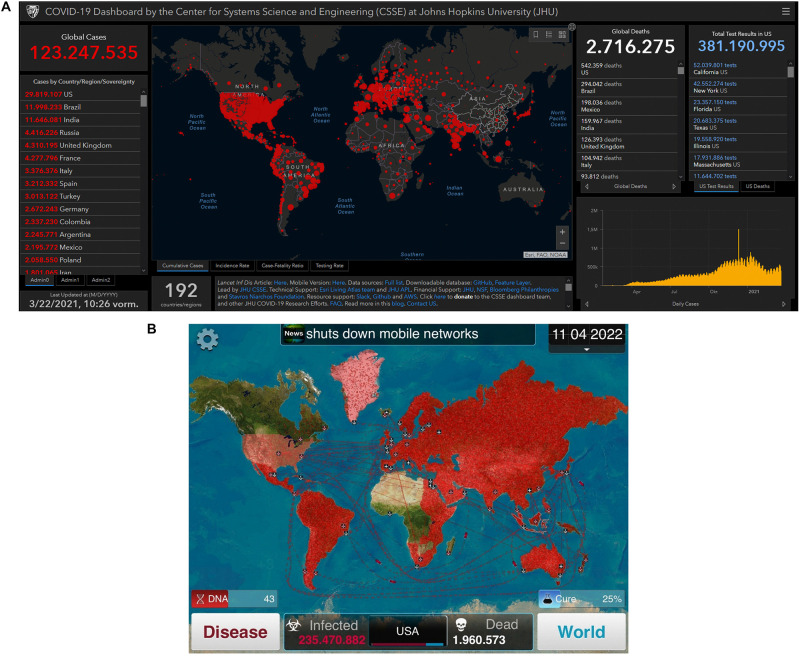
COVID-19 Dashboard by the Center for Systems Science and Engineering (CSSE) at Johns Hopkins University **(A)** as well as the interface of the game “Plague Inc” **(B).**

What is driving people’s behavior right now, in the absence of scripts, concerning the pandemic? We suggest applying the framework of a *homo ludens* as initially described by Huizinga (1949). He proposed *game* and *play* as a way to develop cultural techniques and to make sense of the world. We can apply this mode of understanding—ludification—to COVID-19: Several pandemic-related phenomena bear a resemblance to patterns and mechanisms found in games. This can be explained by analogical reasoning where people detect structural and/or superficial similarities between games (a well-known domain for virtually everybody) and pandemic-related phenomena. In coping with COVID, several characteristic circumstances can be understood in terms of rather diverse game elements:

- **Points:** The use of points–an abstract quantity that can be used to indicate past achievements and failures–is an easy means of gamification (and, as *pointification*, often criticized, for example, by Bogost, 2014). Elaborate data sources like that of the Johns Hopkins Coronavirus Resource and Center (2021) are steadily available and provide 24/7 updated repositories for worldwide infection count and death toll. The graphical interfaces even bear resemblances to games like “Plague Inc” (Ndemic creations, 2012; Figure 1). As many newspapers have integrated those sources into their front web pages, the daily COVID counts are ubiquitous.

- **Leaderboards:** The points mentioned above lend themselves to leaderboards. Deaths (per capita and in total); reproduction number *R_0_* and case fatality rate; the number of tests and number of vaccinations per 100,000 citizens: Those are just some of the most prominent statistics that are used to rank nations, states, or even cities and communities. Usually, they are represented as a line chart to show development over time. Other areas where similar charts are used in the news are highly competitive spheres of life. The finance sector, for example, relies on simple line charts and benchmarks for earnings data and stock share prices. The superficial, visual resemblance of COVID-19 time series and the Dow Jones Industrial Average in the news might make us prone to the fallacy that the driving factors and relations are also similar in some way. And in the worst case, the loss of human life becomes just another index number of economic benchmarks.

- **Roles:** Apart from the protective effect, a mask, and especially the type of face mask, signifies a specific role (cloth face mask vs. N95 vs. face shield) and is associated with the right to access certain areas. Simultaneously, masks disguise the actual bearer, which might contribute to a feeling of deindividuation (Mullen et al., 2003). The intense emotional reactions to mandatory mask usage, in any case, might signify that face coverings have a profound impact on self-awareness. Another role with actual consequences might become available for people who are already vaccinated or have developed antibodies after having gone through COVID-19–those people might be eligible for immunity passports and related privileges (Brown et al., 2020).

- **Collecting Resources:** Some people are hoarders in games. For example, having too much sheep or a pile of ore on one’s hand is rarely a winning strategy in *Settlers of Catan*, as the player becomes vulnerable to game events targeting resource hoarding. Yet, even under those circumstances, excessive collecting is exerted by some players, as it might create an illusion of competence, especially in people with lower ability levels (Kruger and Dunning, 1999), and preparedness (Auf der Heide, 1989; McEntire and Myers, 2004). Likewise, a stockpile of toilet paper will probably not affect one’s chance of surviving COVID-19. In both cases, hoarding probably is a means of maintaining self-efficacy in a complex situation by affecting the belief to withstand difficulties (Bandura, 1990).

- **Archetypes:** Story-rich games often employ archetypes (Jung, 1986; Campbell, 2008) like the *hero*, the *wise old man*, and the *villain*. Archetypes, in their original sense, are basic mental symbols and images that are, according to those theories, rooted in a structure in our mind called *collective unconscious*. While this view is disputed, archetypes can also refer to proto-typical, recurring motifs, and personifications in myth and storytelling that are often superelevated. Campbell’s hero’s journey is the most famous archetypical narrative pattern: an adventurer leaves his home, endures hardship and danger, and return as a transformed person. More often than not, this comes with new abilities and powers. With COVID-19, rituals like *Clap for Carers* indicate archetypical exaggeration: Instead of addressing the real obstacles, like a lack of personal protection equipment (PPE) or bad staff ratios, the personnel’s struggle is romanticized as heroic. In addition, media depictions sometimes reduce key players to some stereotypical quality (e.g., immunologist Anthony Fauci is depicted as a wise counselor, or former U.S. President Donald Trump is portrayed as a villain). In doing so, a complex pandemic runs the danger of being reduced to a personified good-vs.-evil scenario comparable to a campaign in the role-playing game *Dungeons & Dragons*.

- **Location-Based Gaming:** COVID-19 apps have been released in at least 50 nations (McCarthy, 2020). The idea of influencing human behavior by exploiting our awareness for location and direction, with perks and dangers communicated by a spatially aware smartphone, took the world by storm in 2016 with Pokémon Go (Rauschnabel et al., 2017). COVID-19 has made us more spatially aware, and smartphone apps with lock-screen notifications and simple rating indicators inspired by traffic lights have become constant companions for many, especially younger people.

These game mechanics interact dynamically. For example, information obtained from COVID “leaderboards” might influence compliance with preventive measures such as social distancing. Fear is one major aspect regarding this compliance (Harper et al., 2020), and fear might be amplified by yellow and red alerts cast by a COVID app. Hoarding might be a possible consequence, and the resulting empty shelves in the supermarket might be perceived as a signal of impending doom. Likewise, falling infection numbers and good outcome rankings might tempt people to switch from game to play mode and ignore possible dangers while having fun (again).

## The Name of the Game?

All these phenomena are compatible with the idea that COVID-19 can be considered a game–albeit a very serious one. Due to its spatial, temporal, and social expansion, it is a pervasive one (Montola et al., 2009), too.

This should not be taken as an implication that (most) people underestimate the dangers. Falling back on patterns of perception and action from game-related and playful contexts might just be a sign that, in the absence of established societal standards for dealing with a pandemic in North America and Europe, rather generic established patterns–game elements–are applied to make sense of the very real and dangerous threat.

Strictly speaking, we are talking about something analogous to a game here. We do not have more or less definite boundaries between game and real-world in our case (which would be a constituting feature of a game, as discussed by Stenros, 2017). But we argue that there is a benefit in understanding the phenomena described above in terms of game patterns. In a process of analogical reasoning, people might resort to game patterns to draw inferences about the dynamics of a novel, severe and paramount challenge.

However, analogical reasoning might fail when the analogy is derived from superficial similarities and not from structural assumptions—a fallacy common for novices in a domain (Gentner, 1989). Anxiety also increases the risk for employing superficial analogies (Tohill and Holyoak, 2000). How can we improve a game that combines leaderboards, roles, resource collecting, archetypes and spatial awareness? Considering this specific configuration of game elements as a “Game of COVID” allows for strategic optimization of such a game’s mechanics. The elements can then be considered game *design* elements. This deliberate gamification has the potential to shape people’s interaction with their environment. In the best case, such improved dynamics lead to more positive affective and cognitive responses (for a model of the interaction of mechanics, dynamics and esthetics, MDA; see Hunicke et al., 2004).

Leaderboards do motivate people, at least in the short term (Mekler et al., 2013), while introducing a sense of competition (Butler, 2013). Research on the importance of roles has been pioneered by Goffman (1959) and has been described as a theatrical, and thus a somewhat playful act. We can imagine wearing the adequate mask for a given occasion as a performance that is unfamiliar (for most of us), and that is reserved for the public, the “front stage.” Such a behavior is driven extrinsically, not out of conviction. When meeting other people in private, without the need to play a role, masks are likely to be taken down. Regarding the hoarding behavior, we do not know of any studies that examine it in a non-clinical setting. However, a shared experience in famous games like *Settlers of Catan* is: holding resources goes hand in hand with a fear of losing them. The ubiquity of smartphones might add to this low-key feeling of urgency when a COVID tracking app is installed, making the perceived danger a constant and limitless companion.

A board game combining these elements would unfold competitive dynamics, with players holding unfamiliar roles, under a constant feeling of urgency—with a danger that follows us everywhere. This is amplified by metaphors and archetypes that are connected to struggle and eternal combat. Such a rather stressful game would be, like most games, played to win. However, the players would be driven by the fear to lose, feeling detached, without a positive goal, and without a plan B. As a metaphor for a way to deal with a global health hazard, we can imagine that there might be a better way to play.

The game we just sketched, however, seems to us like a rather fitting description for the way many people feel during this pandemic. Borrowing from the game metaphor again, we can now imagine tweaks to this game that are productive and constructive.

## A Rulebook for the Pandemic

Strategic Gamification can build on the spontaneous ludification we have described by changing the game elements to related, but productive and positive design patterns that exploit structural dynamics and not just mere perceptual appearances. The meaning people attribute to behavioral measures in a crisis will change as a result of a more positive dynamic.

- **Leaderboards**: The leaderboards counting people infected, dead, and recovered, are important to assess the status and the trajectory of the pandemic. They dominate the news cycle and emphasize the COVID-19 scores of counties, states and nations. Yet, the pandemic is no competition where the country with the smallest count is the winner. It is not, in contrast to the stock market, a zero-sum game, where the benefit of one is the loss of one another. To shift the focus away from the analogy of a competitive situation, additional metrics that mirror collective effort, mitigation and joint actions should be devised, regularly updated, and should be numerically and graphically added in news distributions. People that are already at a disadvantage or who are discriminated against, are disproportionally affected and at the same time, less visible as sufferers. For them, community action would have the most significant impact (Harris et al., 2020; van Bavel et al., 2020). Besides the number of vaccinations, a dashboard that monitors the number of community groups, the hours spent by volunteers, and the money donated to community initiatives would foster cooperative effort. Instead of line graphs, this might be supported with a visualization akin to node-link-graphs, where accentuated nodes represent engaged community members, and emphasized line edges show strong bonds between those members. This would not only make visible community efforts but could at the same time reinforce cooperative behavior in the direction of “a more communitarian form of conducting ourselves, sharing responsibilities, acknowledging those who are systematically in a position of subalternity, and promoting diversity” (Muriel and Crawford, 2020, p. 154).

- **Roles**: Wearing a mask communicates a special role. It says: I am in a situation that I cannot or will not avoid, and where SARS-CoV-2 transmission is possible. However, it also makes my face, that is, me as a person, less visible. And it acknowledges that there *is* a danger; and masks do not offer perfect protection. At the same time, it is a badge that grants access to areas with a mask mandate, like supermarkets or federal buildings in many Western countries. Instead of being perceived as a barrier for emotional expression (Carbon, 2020) or for virus particles (Chu et al., 2020), the mask needs to become a visible sign for special access rights as well as for a special responsibility. Of course, that is no easy task. The public debate about masks has become highly emotional and polarized. But games—obviously, with role-playing games like *Dungeons & Dragons*, *Gloomhaven* and *Pathfinder*—have always been an opportunity to try out behavior, to handle special responsibilities, and last but not least to explore the perks and burdens of a designated role together with other people/players. Role-playing in games is often built around *playing together* (where other players are at least simulated by a computer as non-playable characters, or are imagined by the vivid descriptions in solo adventures), and this, in turn, has “a unique potential to engage people in collaborative activities” (Zagal et al., 2006, p. 37). When trying to adopt a new role, other people are crucial for us. We can receive feedback and support. Games have the potential to guide this process and to form lasting bonds.

- **Collecting resources**: The importance of civil defense was publicly recognized during the times of the Cold War. In leaflets and phone books, instructions were given on how to react to a crisis. Preparing for food shortages with a stock of durable food, medication, and sanitary products was not considered “prepping” or neurotic, but common-sense behavior. The German Office for Civil Defense and Disaster Recovery has been giving this advice all the time, and the advice is still valid today (Bundesamt für Bevölkerungsschutz und Katastrophenhilfe., 2020). A narrative that sees this stockpiling not as a doomsday measure but as a sensible allocation of resources for the possibility of disrupted services would make *ad hoc* hoarding less necessary. Especially for younger people who did not experience the Cold War situation, the game metaphor might be helpful to acknowledge strategic preparation and to determine the right extent of stockpiling.

- **Archetypes**: Myrick and Willoughby (2021) found that celebrity announcements of a COVID-19 diagnosis, in this case by the Hollywood actor Tom Hanks, can raise awareness of the importance of behavioral changes. Hanks’ diagnosis was of greater impact for those who reported placing high trust in friends, family and celebrities, and those were less likely to be influenced by scientific communication. In terms of Propp’s (1968) narrative elements, the *hero* (Tom Hanks) *struggles* when facing a hazardous event, where an event is a change from one state to another (Rimmon-Kenan, 1983). Hanks’ state has, in public perception, changed from healthy to being ill from a potentially deadly virus. The actor’s journey went on. Tom Hanks has recovered and is now a *hero* that has stood the test in the face of danger. By considering his struggle as a hero’s journey, him prevailing becomes an event in the sense of Slavoj Žižek, not just an occurrence, but “a change of the very frame through which we perceive the world and engage in it” (Žižek, 2014, p. 10). Framing celebrity stories in a way similar to stories described by Campbell (2008) might inspire people to focus not on the dire aspects of infection and illness (and thus, on fear), but on the prospect of persistence and survival (and therefore, hope). The emphasis would also shift from somewhat distant, influential political figures to people that are perceived as relatable and positive. In the long run, this could benefit people with diminished trust in news outlets, public authorities or scientific sources, as “sending of messages about protection measures to the population succeeds not only depends on the choice of means and channel of communication but is decisively influenced by the degree of credibility and trust in the sender” (Robert Koch Institut, 2018, p. 217).

- **Location-Based Gaming**: Right now, the tracking apps show if one *had been* to a place where he or she might have met someone who has been diagnosed with COVID-19 in the time since. A strategic approach would be a mobile app that does not only track and display possible contacts as well as leaderboards for infections, but that makes location-aware suggestions for positive behavior. It might, for example, show recreational areas in the vicinity that are less-visited at the moment. It might also highlight community projects in the area that support elderly and lonely people or families in distress and that are looking for volunteers. Such additions to an existing app would not require the transfer of personal information, so privacy would not be a concern—this will increase the acceptance of such an add-on.

This COVID-game with a revised rulebook would provide us (in addition to the metrics about infections, deaths, and vaccinations) with leaderboards of communal effort and positive change. People are given the opportunity to develop new roles that relate to their personality (instead of just wearing masks as a necessary badge to gain access to supermarkets or federal buildings); roles that build community action. Collecting, storing and distributing stock is not frowned upon, but incentivized as foresighted behavior. Positive role models do not propagate metaphors of a partisan battle but a journey where people recover after hardship. Our smartphones support us by providing real-time location-based opportunities for recreation and volunteering.

The combination of those measures could unfold a dynamic where joint efforts and foresighted behavior become an integral part of people’s roles. This should be supported by success stories of (entertainment as well as local) heroes and by apps that provide real-time information. This game does not negate that the pandemic is a dire situation where millions have died, and many more suffer from lasting symptoms. But it could offer strategic, pro-active opportunities for a situation where many people are struggling for the right way to cope—right now, but also beyond COVID-19.

The strength of game-related approaches in generating a pro-active stance lies in games’ potential to create involvement. Borrowing from Calleja’s model (Calleja et al., 2016), relevant domains are *spatial*, *shared*, *narrative*, *ludic*, *kinesthetic*, and *affective* involvement—all of which can be experienced in immediate engagements in the game (*micro-involvement*) as well as the long-time, off-line involvement (*macro-involvement*). In video games, higher involvement was associated with positive emotions and better learning (Calleja et al., 2016). Other authors stress that digital games can foster introspection (Oliver et al., 2016) and induce meaning (Oliver and Bartsch, 2010). There is a growing number of studies exploring the role games might play in emotion regulation (Hemenover and Bowman, 2018). In other words: a game is the ideal framework where bodily, spatial, social, cognitive, and emotional experiences get intertwined in a motivational experience that affects us beyond the game’s spatio-temporal boundaries.

In the past few months, there have been signs that spontaneous ludification is not a one-way street and that the game’s boundaries are even more permeable. Not only is reality understood in game elements, but elements from reality are spontaneously adopted to enrich games. The game *Minecraft*, which allows for the joint creation of infinitely complex worlds with very simple basic elements (blocks), has not only been used to spread COVID-19-related information and to support other isolated people playing the game. Users have built virtual replicas of hospitals and respiratory centers to teach other users about infectious diseases and their prevention (Hjorth et al., 2020).

## Outlook

One could ask if game design patterns are, in a moral sense, the right way to frame a devastating pandemic. Our rebuttal would be: What would be the right way to frame such a disastrous event? If strategic gamification that builds on spontaneous ludification has the potential to enhance people’s understanding of a situation and to motivate them to protect themselves as well as the people around them, we should consider gamification to be a promising way to generate analogies that help us deal with the pandemic. “Strategic Gamification” identifies spontaneous appearances of game-related mechanisms, deliberately shapes them to improve the dynamics of interaction, intending to foster constructive and positive emotional and cognitive responses–for the current COVID-19 as well as for forthcoming crises.

The present pandemic is an enormous challenge for every public health decision-maker. Exploiting the natural tendency of analogical reasoning by applying game patterns in order to make sense of the world is a promising angle for low-cost and low-threshold health policies. Fighting COVID-19 is not only a game in the domain of medicine, but also of successful communication, of elaboration, of deeper understanding and of insight. The application of strategic gamification has the power to change perception and evaluation, and so might lead to a more reflected form of compliance and trust. Those long-term goals should be the destination for a crisis strategy.

This approach can be applied to other ongoing changes, challenges and crises where people struggle to find appropriate modes of behavior. We could think of the climate change, energy transformation, migration along with integration and inclusion, gentrification, demographic change, or disruptive innovations. These topics are troubled with inactive stakeholders, poor leadership and inadequate or non-strategic plans yielding uncoordinated and hard to understand activities. A strategy to devise sustainable policies should explicitly look for game-related analogies that can be found in the public discourse, or that might be apparent in people’s behavior. Instead of considering what game elements could be introduced, policy makers should try to understand the mechanics, dynamics and even the esthetics that are already present. To achieve this we need psychological investigation and elaborate analyses. Applying game design elements that attract and engage people help to increase acceptance and community feeling.

One very broad distinction regarding games is the separation into competitive (with players having opposing strategies) and cooperative ones (with the possibility for win-win-situations). Zagal et al. (2006) argue for a third category, collaborative, where “all the participants work together as a team, sharing the payoffs and outcomes; if the team wins or loses, everyone wins or loses” (p. 25). If we would try to deliberately take up existing ludic elements (right now mainly competitive ones) and would attempt to gamify the handling of COVID-19, we should aim at introducing metaphors and analogies that emphasize joint effort. Collaborative games, board and video games alike, could be a source of inspiration here. We could evaluate which game design elements are used there to motivate people to work together in order to achieve a common goal. This will enable collaborative behavior and will assist the development of constructive solutions, borne by people, borne for people. These ingredients are essential for sustainable measures accepted by a large majority. There is one very famous board game that can only be mastered when players engage in collaboration. Taking different roles, they have to work together as experts on a map, by sharing crucial information and by exploiting resources to prevail in a dangerous, dynamic world (operationalized via hazard points). The name of the game: Pandemic!

## Data Availability Statement

The original contributions presented in the study are included in the article/supplementary material, further inquiries can be directed to the corresponding author.

## Author Contributions

MHR: original conceptualization, writing – original draft, and revision. NAD: original conceptualization and writing – original draft. C-CC: conceptualization, writing – review and editing, and supervision. All authors contributed to the article and approved the submitted version.

## Conflict of Interest

The authors declare that the research was conducted in the absence of any commercial or financial relationships that could be construed as a potential conflict of interest.
